# A Randomized Placebo‐Controlled Clinical Trial Exploring the Short‐Term Cognitive and Cerebrovascular Effects of Consuming Peppermint Tea: A Mediation Study

**DOI:** 10.1002/hup.70005

**Published:** 2025-04-06

**Authors:** Luka Netzler, Brian Lovell

**Affiliations:** ^1^ Department of Psychology Northumbria University Newcastle Upon Tyne UK

**Keywords:** cerebral blood flow, cognition, integrative medicine, neuropsychiatry, NIRS, peppermint

## Abstract

The cognitive‐enhancing effects of peppermint have been widely reported. Vasodilation, causing an increase in cerebral blood flow (CBF) in the prefrontal cortex, has been implicated as a possible mediator. We tested this here. A total of *N* = 25 individuals, all aged over 18 years, were recruited via convenience sampling. A randomized, single blind placebo‐controlled, independent groups design was used to assess whether groups (peppermint vs. placebo control) could be differentiated with respect to change in cognition, assessed via a computerized battery, and change in cerebral blood flow, assessed with Near‐Infrared‐Spectroscopy (NIRS), from pre‐post intervention. Groups disparities in both cognitive and cerebrovascular change scores (from pre‐post intervention) emerged. Improvements in cognitive performance were better in the peppermint group. Increases in hemodynamic activity, indexed by Oxygenated (Oxy‐Hb) and Total hemoglobin (Total‐Hb), were also greater in the peppermint group. Cerebrovascular changes from pre‐to post‐intervention were unrelated to cognitive changes over the same period, ruling out mediation effects. In conclusion, 200 mL of peppermint, consumed as tea, effectively boosted cognition and cerebral blood flow in otherwise healthy adults. Increased cerebral blood flow, however, did not mediate the cognitive‐enhancing effects of peppermint. Future research incorporating larger samples and exploring other neurophysiological mediators is encouraged.

## 1 Introduction

Peppermint, consumed orally or inhaled, has been shown to have cognitive‐enhancing properties. For example, Moss et al. (2008) found that cognition, assessed via attention and memory, improved following the inhalation of peppermint aroma. No changes were observed in placebo controls. Several other studies have reported similar positive changes in cognitive functions, especially attention and memory, following the consumption and inhalation of peppermint (Barbalho et al. 2011; Meamarbashi 2014). Most recently, using a sample of cognitively healthy undergraduate students, Abdelhalim (2021) observed improvements in both retroactive and prospective memory following the inhalation of a peppermint aroma. Each of these studies recommended that future researchers endeavor to identify the mechanism, neurophysiological or otherwise, by which peppermint consumption translates into acute cognitive enhancement.

Menthol, one key ingredient in peppermint, is a known acetylcholine receptor (nAChR) agonist, inhibiting acetylcholinesterase and preventing acetylcholine reuptake (López et al. 2010; McCombs et al. 2011; Orhan et al. 2008). Acetylcholine has been linked with cognitive functions such as memory recall and consolidation, making it one viable pathway by which peppermint could improve cognitive performance (Kennedy et al. 2018; López et al. 2010; McCombs et al. 2011).

Others have posited that menthol could lead to positive cerebrovascular changes, increasing levels of oxygenated blood in the brain (Doss and Nagarajan 2023; Jasira et al. 2013; Silva 2020). Indeed, increased cerebral blood flow (CBF), by making oxygen and glucose more readily available, would likely facilitate the synthesis of neurochemicals such as acetylcholine, positively impacting cognition (Brekke et al. 2015; Dukart et al. 2018). This effect might be particularly salient in the prefrontal cortex (PFC), an area known to regulate executive functions such as working memory and attention (Casey et al. 2001; Friedman and Robbins 2021; Haber et al. 2021). Furthermore, increased cerebral blood flow supports the clearing of metabolic waste from the PFC, potentially enhancing the cognitive benefits of consuming vasodilatory compounds such as peppermint (Benveniste et al. 2018).

Near‐Infrared Spectroscopy (NIRS) is one well‐established, non‐invasive technique for measuring CBF. Its portability, ease of use, and low cost make NIRS the preferred imaging technique for many researchers (Jackson and Kennedy 2013). Moreover, strong correlations have been reported between NIRS data and data collected with other imaging techniques such as positron emission tomography (ET) (Cui et al. 2011; Dukart et al. 2018). NIRS quantifies concentrations of oxygenated hemoglobin (Oxy‐Hb), deoxygenated hemoglobin (Deoxy‐Hb), and total hemoglobin (Total‐Hb) in the brain. These hemodynamic signals are often used as proxies for neural activity (Ayaz et al. 2019; Sato et al. 2013). A plethora of studies have used NIRS to assess the potential cerebrovascular impact of consuming vasoactive compounds such as caffeine and resveratrol (Higashi et al. 2004; Kennedy et al. 2010). NIRS, therefore, is a well‐accepted and widely used technique for assessing CBF in the context of nutritional neuroscience.

Researchers have posited that peppermint consumption, because it increases blood flow to the prefrontal cortex, improves cognitive functions like attention and memory. Here, we test this formally. We hypothesized that improvements in (a) cognition, assessed via executive function tasks (memory, attention), and (b) increases in cerebral blood flow, assessed via NIRS, will be observed following consumption of peppermint. It is further hypothesized that (c) cerebrovascular changes following peppermint consumption will predict changes in cognition over the same period and that (d) the positive cognitive effects of peppermint consumption will be mediated by increased cerebrovascular activity.

## 2 Methods

### 2.1 Design and Participants

The study used a single blind randomized, placebo‐controlled independent groups design to compare change scores in cognition and cerebrovascular function between the experimental (peppermint) and control (placebo) groups.

A sample of *N* = 25 participants (mean age = 30.52 ± 11.54, range 20–56, 52% female) was recruited via convenience, matching or exceeding the sample sizes of several studies asking similar questions (Ide et al. 2014; Kennedy et al. 2018; Meamarbashi and Rajabi 2013). Participants were recruited according to strict criteria: (a) aged > 18 years, (b) no diagnosed neurological or mental health conditions, (c) no diagnosed cognitive impairment, and (d) no self‐reported use of recreational drugs. Participants were randomized using computer‐generated random numbers into two groups: experimental (peppermint) and control (placebo).

### 2.2 Measures

#### 2.2.1 Cognitive Assessment

Cognition was assessed with the Computerized Mental Performance Assessment System (COMPASS). Previous research has shown that peppermint consumption positively impacts executive functions such as working and episodic memory, and attention (Abdelhalim 2021; Moss et al. 2016). Neural correlates of executive function performance have been widely studied, with researchers observing consistent activation in prefrontal regions (Friedman and Robbins 2021; Haber et al. 2021). Therefore, executive function tasks—working/episodic memory and attention—were the focus here. The cognitive tasks selected have been linked with prefrontal cortical activity and have been shown to be sensitive to peppermint consumption (Deivanayagame et al. 2020; Hoult et al. 2019; Scholey et al. 2010).

#### 2.2.2 Task One: Picture Recall

This task assesses episodic memory. It shows participants 30 random images for one minute. Following this, participants are shown the same 30 images mixed with several ‘distractor’ images. Participants are asked whether each image they see is one they saw previously, pressing Y for Yes and N for No on their keyboard. Correct responses range from 0 to 30, with higher scores indicative of better episodic memory.

#### 2.2.3 Task Two: Serial Threes

This task assesses attention and working memory. Participants are provided with a random number between 800 and 900 and then asked, for one minute, to keep subtracting three, recording their answer using the keyboard. The number of correct responses is summed, with higher scores reflecting better working memory and executive control.

#### 2.2.4 Task Three: Word Recall

This task assesses short‐term memory (STM). Participants are shown 16 words total. Words are presented at a rate of one per second. Participants are then asked to recall as many of the words as possible in 1 minute. The number of correctly recalled words is summed. Higher scores indicate better STM.

#### 2.2.5 Task Four: Corsi Blocks

This task assesses visuospatial working memory. Participants are presented with several squares (i.e., blocks) on the screen. The blocks are then illuminated in a sequence. Participants are asked, using their keyboard, to recall the correct sequence of illumination. Illumination sequences become harder (i.e., strings of illuminated boxes become longer) with more correct responses, continuing until participants have made a total of three errors. Participants' total score is calculated as the average number of blocks recalled across all correctly identified sequences. Higher scores represent better visuospatial working memory.

### 2.3 Protocol

Participants were randomized to the experimental (peppermint) or control (placebo) condition using computer‐generated random numbers. Participants allocated to the peppermint condition received 200 mL of peppermint tea (Heath & Heather) brewed for 5 minutes. Dosage was informed by the protocol of Moss et al. (2008). Participants in the control condition received 200 mL of warm water. They were told the warm water contained a tasteless and flavorless peppermint extract. This deception was necessary to safeguard the robustness of the protocol, ensuring that anticipatory effects were comparable in both groups and to rule out non‐specific effects derived from consuming a warm drink.

Participants in each condition were asked to wait for 20 min following consumption. This absorption period was informed by previous studies that assessed the cognitive effects of consuming vasodilatory compounds (Hindmarch et al. 1998; Moss et al. 2008). Moreover, it is well established that it takes approximately 20 min for fluids to move from the small intestine into the bloodstream (Péronnet et al. 2011).

### 2.4 Near‐Infrared Spectroscopy (Cerebral Blood Flow)

The Oxymon 2‐channel continuous wave system, developed by Artinis Medical Systems (BV, Zetten, Netherlands), was used to assess cerebral blood flow. Relative changes in the absorption of infrared light were measured at a time resolution of 10 Hz, with hemoglobin readings reported in micromolar units (μM). Concentrations of Oxy‐Hb, Deoxy‐Hb, and Total‐Hb were calculated using a modified Beer–Lambert law based on the absorption of infrared light at 764 and 856 nm (Duncan et al. 1996; Kamran et al. 2018; Obrig and Villringer 2003; Talukdar et al. 2013).

Optodes, placed 35 mm apart, were placed on participants forehead at the midpoint of the Fp1 and F3 landmarks for the left prefrontal cortex, and the midpoint of the Fp2 and F4 landmarks for the right prefrontal cortex, based on the international 10–20 EEG system by Jasper (1958). Optode distance and placement used here are regarded as optimal for the measurement of hemodynamic activity (Wang et al. 2019). Optodes were attached to the skin with self‐adhesive disks, and a black elastic strap was worn around the head to secure them and to reduce motion artifacts.

Optode readings revealed that Oxy‐Hb and Deoxy‐Hb, averaged across all study time points, was comparable across left and right hemispheres (all *p*s > 0.44). Therefore, in keeping with other studies asking similar questions, these cerebrovascular indices were averaged across hemispheres at all study time points (Moss et al. 2008).

### 2.5 Procedure

Participants were invited to our lab on a day and time suitable for them. Consent contingent, participants were randomized to the peppermint (experimental) or placebo (control) group. They were then fitted with the NIRS headband. Resting cerebrovascular activity was established for 2 minutes. Participants were then invited to complete several computerized cognitive tasks using COMPASS. Cerebral blood flow was continuously measured throughout. Following completion of the cognitive tasks, participants randomized to the experimental group were given a cup of peppermint (200 mL) tea. Controls received 200 mL of warm water infused, they were told, with a tasteless and odorless peppermint extract. Participants were asked to wait for 20 min to allow for absorption before completing the same cognitive tests for a second time. Completion of the post‐intervention assessments of cognition and cerebrovascular activity was followed by a debrief.

**FIGURE 1:**
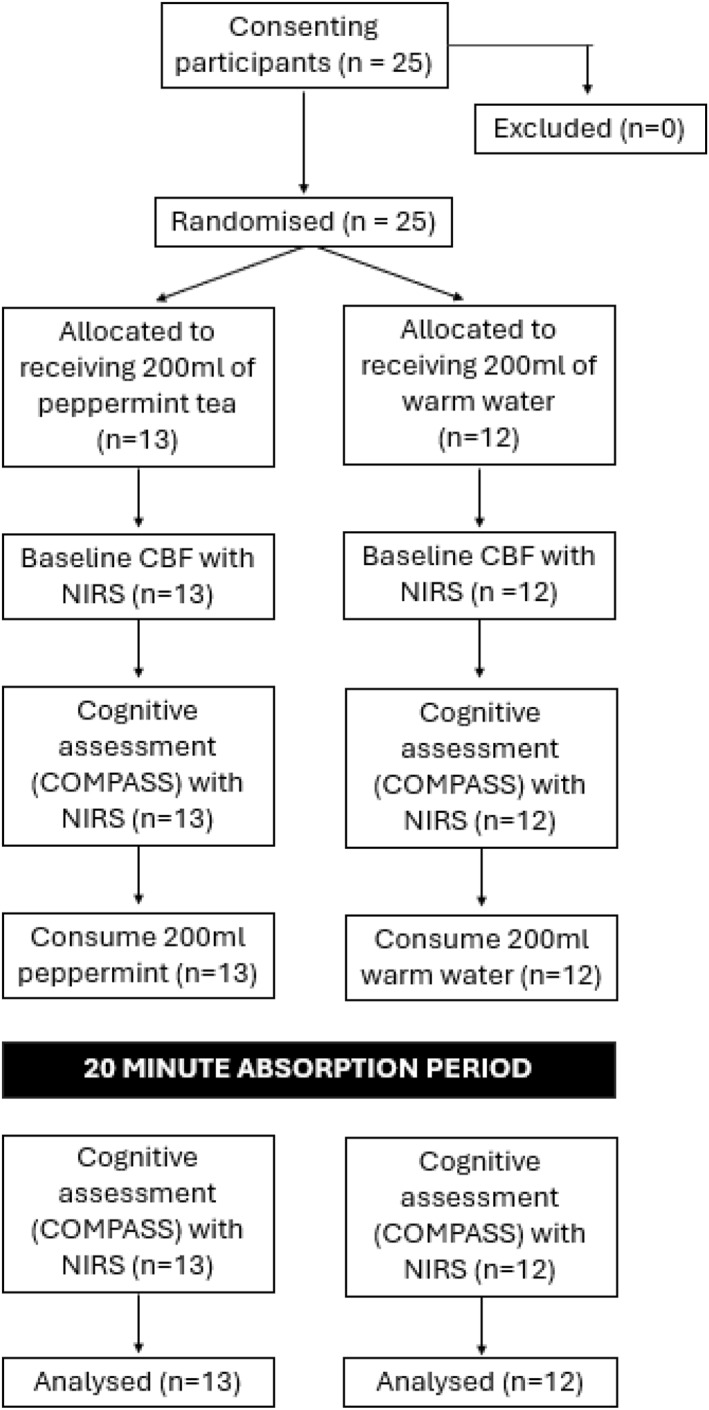
Flow diagram of all participants throughout study phases.

The study and all its procedures were risk assessed and approved by the institutional, Faculty of Health and Life Sciences ethics committee. The study was done in accord with the principles stated in the Declaration of Helsinki, with all participants providing fully informed consent to take part. Figure 1 displays a CONSORT flowchart.

### 2.6 Treatment of Data

A series independent *t* tests and, for categorical data, chi‐square tests (*χ* ^*2*^) were used to compare groups with respect to socio‐demographic and lifestyle data.

The cognitive tasks used here were highly correlated in previous research (Dede et al. 2015). As such, a one‐way multivariate analysis of variance (MANOVA) was used to explore whether groups (peppermint and placebo control) might be differentiated with respect to a composite cognitive change score. This was calculated as the average change across all four cognitive tasks from pre to post consumption. A significant result was followed up with a series of one‐way, Bonferroni corrected ANOVAs to explore whether groups might be distinguished with respect to changes in individual cognitive indices.

Absolute measurements are not possible with NIRS; it is only possible to establish hemodynamic response (i.e., change). As such, resting hemodynamic activity, captured via Oxy‐Hb, Deoxy‐Hb, and Total‐Hb, was subtracted from hemodynamic activity during completion of cognitive tasks at pre‐and post‐consumption. This resulted in two measures of cerebrovascular activity: (a) change in hemodynamic activity between resting and cognitive task completion pre‐consumption, and (b) change in hemodynamic activity between resting and cognitive task completion post‐intervention. Pre‐intervention change was then subtracted from post‐intervention change to capture participants' overall cerebrovascular change from pre to post intervention. One‐way MANOVA was used to explore possible group differences with respect to a composite cerebrovascular change score from pre‐post consumption. This was followed up with a series of one‐way, Bonferroni corrected ANOVAs assessing potential group disparities with respect to change in individual hemodynamic parameters.

Bivariate correlation was used to assess whether cognitive change scores from pre‐post consumption were related to change scores in cerebrovascular activity across the same period. Mediation effects were assessed via one‐way ANCOVA, establishing whether group disparities with respect to cognitive change scores from pre‐post consumption might be lost after adjustment for cerebrovascular change scores across the same period. Indeed, for mediation to occur, as per Baron and Kenny (1986), the independent variable (IV), group (peppermint vs. control), needs to predict both the mediator (cerebrovascular change) and the outcome (cognitive change). Moreover, the mediator, after adjusting for the IV, needs to predict the outcome, and the relationship between the IV and outcome, after adjusting for the mediator, ought to be substantially reduced and, for full mediation, non‐significant.

## 3 Results

### 3.1 Assumption Checking

Kolmogorov‐Smirnov tests revealed that cognitive change scores, averaged across cognitive tasks, were normally distributed (*D* (25) = 0.11, *p* = 0.20). This was also the case for change scores for each of the cerebrovascular indices, oxy‐HB, (*D* (25) = 0.14, *p* = 0.19), deoxy‐HB (*D* (25) = 0.14, *p* = 0.20), and total‐HB (*D* (25) = 0.20, *p* = 0.20). No data transformation was needed.

### 3.2 Socio‐Demographic and Lifestyle Characteristics

Independent *t* tests revealed that groups were comparable with respect to age (*t* (23) = −0.03, *p* = 0.98), number of cigarettes smoked per day (*t* (23) = 1.33, *p* = 0.96), alcohol intake (drinks) per week (*t* (23) = −1.73, *p* = 0.09), and average hours of sleep per night (*t* (23) = −1. 62, *p* = 0.12). A series of chi square tests revealed groups were also indistinguishable on gender (*χ* ^2^ (2) = 2.47, *p* = 0.29), highest level of education (*χ* ^2^ (4) = 5.66, *p* = 0.23), and relationship status (*χ* ^2^ (1) = 2.49, *p* = 0.11). Therefore, none of these variables were controlled in subsequent analyses.

### 3.3 Cognitive and Cerebrovascular Change

The MANOVA revealed a significant effect of group on the composite cognitive change score (Wilks' Lambda = 0.083, *F* (4, 20) = 55.20, *p* < 0.001). This was followed up with a series of Bonferroni‐corrected univariate ANOVAs, with *p*‐values < 0.012 now deemed significant. For all four cognitive tasks, picture recall (*F* (1, 23) = 13.66, *p* < 0.001), serial subtraction (*F* (1, 23) = 7.70, *p* = 0.011), Corsi‐Block (*F* (1, 23) = 18.52, *p* < 0.001), and word recall (*F* (1, 23) = 93.90, *p* < 0.001), the magnitude of change (i.e., cognitive improvement) was greater in the peppermint group.

A second MANOVA, assessing whether groups might be differentiated on composite cerebrovascular change from pre‐post consumption, yielded a significant effect of group (Wilks' Lambda = 0.083, *F* (4, 20) = 55.20, *p* < 0.001). This was followed up with a series of Bonferroni‐corrected univariate ANOVAs, with *p*‐values < 0.017 now deemed significant. Group disparities emerged with respect to changes in Oxy‐Hb (*F* (1, 23) = 16.74, *p* < 0.001) and Total‐Hb (*F* (1, 23) = 8.76, *p* = 0.007); increases in hemodynamic activity were greater in the peppermint condition. Groups were comparable with respect to change in Deoxy‐Hb (*F* (1, 23) = 0.55, *p* = 0.466). Table 1 displays means and standard deviations for all outcomes of interest by group.

**TABLE 1:** Means and standard deviations for cognitive and cerebrovascular change scores by group.

Change scores	Treatment (*N* = 13)		Control (*N* = 12)		*p*
	*M*	SD	*M*	SD	
Picture recall	3.33	3.33	−1.11	2.60	0.001
Serial subtraction	6.00	5.10	1.17	3.40	0.011
Word recall	2.38	0.90	−0.42	0.52	< 0.001
Corsi block task	1.31	1.01	−0.09	0.50	< 0.001
Oxy‐Hb	1.53	1.29	−0.78	1.53	< 0.001
Deoxy‐Hb	−0.04	1.62	0.37	1.06	0.466
Total‐Hb	1.47	1.15	−0.44	2.00	0.007

Abbreviations: Deoxy‐Hb = Deoxygenated haemoglobin, Oxy‐Hb = Oxygenated haemoglobin, Total‐Hb = Total haemoglobin.

### 3.4 Mediation Effects

Bivariate correlation revealed no relationship between cognitive change scores, assessed as a composite or as individual cognitive indices, and cerebrovascular change scores, assessed as Oxy‐Hb and Total‐Hb, from pre‐post consumption (all *p*s > 0.11). Moreover, after adjusting for cerebrovascular change, assessed as change in Oxy‐Hb and Total‐Hb, group disparities in cognitive change, assessed as a composite and as individual cognitive indices, remained significant (all *p*s < 0.05). It would appear the beneficial effects of peppermint consumption are not mediated by changes in cerebral blood flow.

## 4 Discussion

We sought to investigate the effects of peppermint consumption on cognitive performance and cerebral blood flow (CBF). We were particularly interested in whether any beneficial cognitive effects of peppermint consumption might be mediated by increased prefrontal hemodynamic activity.

Findings revealed that improvements in cognition, particularly memory (episodic and working) and attention, were greater in the peppermint condition compared with placebo controls. Peppermint consumption was also associated with greater hemodynamic change, captured via Oxy‐Hb and Total‐Hb, in the prefrontal cortex (PFC). However, cognitive change following peppermint consumption was unrelated to cerebrovascular change across the same period, ruling out mediation effects. Therefore, the adaptive cognitive effects of peppermint consumption in tea do not appear to be mediated by increased prefrontal hemodynamic activity.

Finding that peppermint improves cognition, particularly in domains such as memory and attention, is commensurate with several previous studies. Moss et al. (2008) observed positive changes in memory and subjective attention, and Abdelhalim (2021) observed improvements in several memory domains, following the inhalation of peppermint. The current study extends these findings, showing that oral peppermint supplementation at a relatively low dose (200 mL) engenders positive cognitive changes. Closer inspection of the data shows that controls performed poorer on some cognitive tasks (picture and word recall) following the consumption of warm water (i.e., placebo), generating negative change scores. It might be the case that cognitive fatigue, often experienced when completing cognitive tasks multiple times in a short span of time, is negated with peppermint supplementation, explaining the results observed here (Kazemi et al. 2024). Peppermint consumption positively impacted multiple cognitive indices here. However, improvements in visuo‐spatial memory, assessed via Corsi blocks, was a particularly noteworthy finding as this has not been previously explored in human studies.

The observed increases in Oxy‐Hb and Total‐Hb following consumption of peppermint are consistent with the well‐established vasodilatory effects of menthol. Closer inspection of the data demonstrates that Oxy‐Hb and Total‐Hb increased from pre‐post consumption in the peppermint, but not in the control condition. Given that peppermint consumption improves cognition, and that cognitive performance largely depends on the supply of oxygenated blood, it would be reasonable to hypothesize that peppermint enhances cognitive performance through its vasodilatory effects, increasing the delivery of metabolic substrates to the prefrontal cortex. However, mediation testing did not support this. That is, cognitive change, assessed as both a composite and as individual cognitive indices, was unrelated to cerebrovascular change scores from pre‐post intervention. Moreover, group differences in cognitive change scores remained after adjusting for cerebrovascular change scores. Therefore, it would appear the adaptive cognitive changes observed following peppermint consumption are not mediated by increased prefrontal hemodynamic activity. That said, mediation might be obscured by the small sample size (*N* = 25). Indeed, mediation models generally need more than 50 participants to detect indirect effects, especially when effect sizes are modest (Fritz and MacKinnon 2007; Sim et al. 2022). Alternative mediating pathways, especially neurochemical modulators, should not be ruled out. Indeed, menthol acts as a positive allosteric modulator of acetylcholine receptors (nAChRs), enhancing cholinergic neurotransmission critical for cognitive processes such as memory and attention (López et al. 2010). Additionally, menthol inhibits acetylcholinesterase, the enzyme responsible for degrading acetylcholine, further promoting cholinergic activity (Kennedy et al. 2018; Orhan et al. 2008). Moreover, by activating transient receptor potential melastatin 8 (TRPM8) channels, menthol can induce a cooling sensation, increasing alertness via changes in neuronal excitability (; Wei 2020). These neurochemical pathways might play a significant role in cognitive enhancement, and this should be explored in future work.

Findings reported here suggest that peppermint serves as one simple, natural intervention to enhance cognitive function, particularly in tasks requiring memory and attention. This might have applications in educational and occupational environments where cognitive demand tends to be higher. Larger samples are needed in future work, as is a granular compound analysis, to help identify which bioactive components contribute most readily to the cognitive and vascular effects of peppermint consumption. Indeed, peppermint contains multiple compounds such as menthol, menthone, and limonene (Kamatou et al. 2013). Isolating the effects of these compounds individually might help better elucidate their roles in modulating neural activity and cerebrovascular change. Moreover, incorporating neuroimaging methods with higher spatial resolution would offer more precise measurements of CBF and neural activation, helping researchers determine whether changes in other cortical locations and neurotransmitter‐related systems might be driving the positive cognitive impact of peppermint.

These findings, showing the cognitive‐enhancing effects of small doses of peppermint consumed in tea, could also have clinical implications. Use of phytotherapy has become popular in various clinical groups, reflecting a growing trend for integrative medicine. For example, it was estimated by Hashempur et al. (2018) that > 77% of patients with dyslipidemia, a condition of unhealthy levels of lipids, used medicinal herbs, like peppermint, for their potential palliative effects. Several studies recently reported how integrative medicine, inclusive of medicinal herbs, produced clinical benefits, reducing respiratory problems following COVID‐19 infection and facilitating positive changes for those with metabolic syndrome (Ahmed et al. 2022; Nawrot et al. 2022). The clinical gains of phytotherapy have also been captured in recent review articles (Salm et al. 2023).

Future studies might consider whether the cognitive‐enhancing properties of peppermint, as was observed here, underlie clinical gains for certain populations. For example, it is well known that patients with neurodegenerative conditions, like dementia, often experience clinically elevated levels of depression (Cipriani et al. 2015). Depression has been shown to be sensitive to cognitive decline, with faster cognitive decline predicting increased depressive symptomology among otherwise healthy adults (Li et al. 2025) and those with clinically verified neurocognitive disorders such as dementia (Rapp et al. 2011). Medicinal plants, like peppermint, through their influence of neurochemicals, like serotonin, might arrest cognitive decline in the short term, providing temporary emotional gains for clinical populations. Future studies might explore this possibility.

Findings reported here should be considered in the context of several study limitations. First, using an independent groups design, as opposed to a within‐subjects design, may have introduced individual differences that affected the outcomes. However, despite the small sample size, groups were comparable on all socio‐demographic and lifestyle data, suggesting randomization was effective. That said, diet, caffeine, and physical activity were not measured here, and each of these variables has been shown to be influential for cognition and cerebrovascular change (Higashi et al. 2004; Kennedy and Haskell 2011). This was a noteworthy limitation that should be addressed in future work. In addition, the simple pre‐post design used here precludes us from establishing the longevity of the positive cognitive changes observed following peppermint consumption. Whether these positive effects are sustainable, lasting for hours or days, should be explored in future work.

In conclusion, consuming 200 mL of peppermint tea was effective for improving performance in several cognitive areas, most notably memory and attention. Cerebrovascular changes, manifesting as increases in Oxy‐Hb and Total‐Hb, were also observed following peppermint consumption. However, cerebrovascular change scores were unrelated to cognitive change scores, ruling out increased hemodynamic activity as one possible determinant for improved cognition following peppermint consumption. Studies recruiting larger samples and that consider alternative neural and cerebrovascular mediators are warranted.

## Conflicts of Interest

The authors declare no conflicts of interest.

## Data Availability

Data are available in electronic repository and can be made available on request.

## References

[hup70005-bib-0001] Abdelhalim, A. R. 2021. “The Effect of Mentha Piperita L. On the Mental Health Issues of University Students: A Pilot Study.” Journal of Pharmacy & Pharmacognosy Research 9, no. 1: 49–57. 10.56499/jppres20.932_9.1.49.

[hup70005-bib-0002] Ahmed, M. , N. Kumari , Z. Mirgani , et al. 2022. “Metabolic Syndrome; Definition, Pathogenesis, Elements, and the Effects of Medicinal Plants on its Elements.” Journal of Diabetes and Metabolic Disorders 21, no. 1: 1011–1022. 10.1007/s40200-021-00965-2. 35673459 PMC9167315

[hup70005-bib-0003] Ayaz, H. , M. Izzetoglu , K. Izzetoglu , and B. Onaral . 2019. The Use of Functional Near‐Infrared Spectroscopy in Neuroergonomics. Elsevier eBooks. 10.1016/b978-0-12-811926-6.00003-8.

[hup70005-bib-0004] Barbalho, S. M. , D. C. Damasceno , A. P. M. Spada , et al. 2011. “Metabolic Profile of Offspring From Diabetic Wistar Rats Treated With Mentha Piperita (Peppermint).” Evidence‐based Complementary and Alternative Medicine 2011, no. 1. 10.1155/2011/430237. PMC3106428 21647314

[hup70005-bib-0005] Baron, R. M. , and D. A. Kenny . 1986. “The Moderatorâ–Mediator Variable Dist Inction in Social Psychological Research: Conceptual, Strategic, and Statistical Considerations.” Journal of Personality and Social Psychology 51, no. 6: 1173Â–1182. 10.1037/0022-3514.51.6.1173. 3806354

[hup70005-bib-0006] Benveniste, H. , X. Liu , S. Koundal , S. Sanggaard , H. Lee , and J. Wardlaw . 2018. “The Glymphatic System and Waste Clearance With Brain Aging: A Review.” Gerontology 65, no. 2: 106–119. 10.1159/000490349. 29996134 PMC6329683

[hup70005-bib-0007] Brekke, E. , T. S. Morken , and U. Sonnewald . 2015. “Glucose Metabolism and Astrocyte–Neuron Interactions in the Neonatal Brain.” Neurochemistry International 82: 33–41. 10.1016/j.neuint.2015.02.002. 25684072

[hup70005-bib-0008] Casey, B. J. , S. D. Forman , P. Franzen , et al. 2001. “Sensitivity of Prefrontal Cortex to Changes in Target Probability: A Functional MRI Study.” Human Brain Mapping 13, no. 1: 26–33. 10.1002/hbm.1022. 11284044 PMC6871821

[hup70005-bib-0009] Cipriani, G. , C. Lucetti , C. Carlesi , S. Danti , and A. Nuti . 2015. “Depression and Dementia. A Review.” European Geriatric Medicine 6, no. 5: 479–486. 10.1016/j.eurger.2015.07.010.

[hup70005-bib-0010] Cui, X. , S. Bray , D. M. Bryant , G. H. Glover , and A. L. Reiss . 2011. “A Quantitative Comparison of NIRS and fMRI Across Multiple Cognitive Tasks.” NeuroImage 54, no. 4: 2808–2821. 10.1016/j.neuroimage.2010.10.069. 21047559 PMC3021967

[hup70005-bib-0011] Dede, E. , I. Zalonis , S. Gatzonis , and D. Sakas . 2015. “Integration of Computers in Cognitive Assessment and Level of Comprehensiveness of Frequently Used Computerized Batteries.” Neurology Psychiatry and Brain Research 21, no. 3: 128–135. 10.1016/j.npbr.2015.07.003.

[hup70005-bib-0012] Deivanayagame, B. , A. V. Kumar , K. N. Maruthy , and S. Kareem . 2020. “Effect of Peppermint Aroma on Short Term Memory and Cognition in Healthy Volunteers.” International Journal of Physiology. 10.37506/ijop.v8i1.10.

[hup70005-bib-0013] Doss, V. A. , and A. Nagarajan . 2023. “Antihypertrophic Effect of Menthol From Mentha X Piperita Concerning Cardiac Hypertrophy: A Review.” Natural Products Journal 13, no. 2. 10.2174/2210315512666220429110704.

[hup70005-bib-0014] Dukart, J. , Š. Holiga , C. Chatham , et al. 2018. “Cerebral Blood Flow Predicts Differential Neurotransmitter Activity.” Scientific Reports 8, no. 1: 4074. 10.1038/s41598-018-22444-0. 29511260 PMC5840131

[hup70005-bib-0015] Duncan, A. , J. H. Meek , M. Clemence , et al. 1996. “Measurement of Cranial Optical Path Length as a Function of Age Using Phase Resolved Near Infrared Spectroscopy.” Pediatric Research 39, no. 5: 889–894. 10.1203/00006450-199605000-00025. 8726247

[hup70005-bib-0016] Friedman, N. P. , and T. W. Robbins . 2021. “The Role of Prefrontal Cortex in Cognitive Control and Executive Function.” Neuropsychopharmacology 47, no. 1: 72–89. 10.1038/s41386-021-01132-0. 34408280 PMC8617292

[hup70005-bib-0017] Fritz, M. S. , and D. P. MacKinnon . 2007. “Required Sample Size to Detect the Mediated Effect.” Psychological Science 18, no. 3: 233–239. 10.1111/j.1467-9280.2007.01882.x. 17444920 PMC2843527

[hup70005-bib-0018] Haber, S. N. , H. Liu , J. Seidlitz , and E. Bullmore . 2021. “Prefrontal Connectomics: From Anatomy to Human Imaging.” Neuropsychopharmacology 47, no. 1: 20–40. 10.1038/s41386-021-01156-6. 34584210 PMC8617085

[hup70005-bib-0019] Hashempur, M. H. , S. H. Mosavat , M. Heydari , and M. Shams . 2018. “Medicinal Plants' Use Among Patients With Dyslipidemia: An Iranian Cross‐Sectional Survey.” Journal of Complementary and Integrative Medicine 16, no. 3. 10.1515/jcim-2018-0101. 30391934

[hup70005-bib-0020] Higashi, T. , Y. Sone , K. Ogawa , et al. 2004. “Changes in Regional Cerebral Blood Volume in Frontal Cortex During Mental Work With and Without Caffeine Intake: Functional Monitoring Using Near‐Infrared Spectroscopy.” Journal of Biomedical Optics 9, no. 4: 788. 10.1117/1.1755233. 15250767

[hup70005-bib-0021] Hindmarch, I. , P. T. Quinlan , K. L. Moore , and C. Parkin . 1998. “The Effects of Black Tea and Other Beverages on Aspects of Cognition and Psychomotor Performance.” Psychopharmacology 139, no. 3: 230–238. 10.1007/s002130050709. 9784078

[hup70005-bib-0022] Hoult, L. , L. Longstaff , and M. Moss . 2019. “Prolonged Low‐Level Exposure to the Aroma of Peppermint Essential Oil Enhances Aspects of Cognition and Mood in Healthy Adults.” American Journal of Plant Sciences 10, no. 06: 1002–1012. 10.4236/ajps.2019.106072.

[hup70005-bib-0023] Ide, K. , H. Yamada , N. Takuma , et al. 2014. “Green Tea Consumption Affects Cognitive Dysfunction in the Elderly: A Pilot Study.” Nutrients 6, no. 10: 4032–4042. 10.3390/nu6104032. 25268837 PMC4210905

[hup70005-bib-0024] Jackson, P. A. , and D. O. Kennedy . 2013. “The Application of Near Infrared Spectroscopy in Nutritional Intervention Studies.” Frontiers in Human Neuroscience 7. 10.3389/fnhum.2013.00473. PMC3741577 23964231

[hup70005-bib-0025] Jasira, M. , K. Sai‐Sailesh , and J. K. Mukkadan . 2013. “Oral Administration of Peppermint in Wistar Albino Rats: Memory Boosting and Regaining.” Indonesian Journal of Biomedical Sciences 7, no. 1. 10.15562/ijbs.v7i1.109.

[hup70005-bib-0026] Jasper, H. 1958. “Report of the Committee on Methods of Clinical Examination in Electroencephalography.” Electroencephalography and Clinical Neurophysiology 10, no. 2: 370–375. 10.1016/0013-4694(58)90053-1.

[hup70005-bib-0027] Kamatou, G. P. P. , I. Vermaak , A. M. Viljoen , and B. M. Lawrence . 2013. “Menthol: A Simple Monoterpene With Remarkable Biological Properties.” Phytochemistry 96: 15–25. 10.1016/j.phytochem.2013.08.005. 24054028

[hup70005-bib-0028] Kamran, M. A. , M. M. N. Mannann , and M. Y. Jeong . 2018. “Differential Path‐Length Factor’s Effect on the Characterization of Brain’s Hemodynamic Response Function: A Functional Near‐Infrared Study.” Frontiers in Neuroinformatics 12. 10.3389/fninf.2018.00037. PMC6019851 29973875

[hup70005-bib-0029] Kazemi, A. , A. Iraji , N. Esmaealzadeh , M. Salehi , and M. H. Hashempur . 2024. “Peppermint and Menthol: A Review on Their Biochemistry, Pharmacological Activities, Clinical Applications, and Safety Considerations.” Critical Reviews in Food Science and Nutrition 65, no. 8: 1–26. 10.1080/10408398.2023.2296991. 38168664

[hup70005-bib-0030] Kennedy, D. O. , and C. F. Haskell . 2011. “Cerebral Blood Flow and Behavioural Effects of Caffeine in Habitual and Non‐habitual Consumers of Caffeine: A Near Infrared Spectroscopy Study.” Biological Psychology 86, no. 3: 298Â–306. 10.1016/j.biopsycho.2010.12.010. 21262317

[hup70005-bib-0031] Kennedy, D. O. , E. Okello , P. L. Chazot , et al. 2018. “Volatile Terpenes and Brain Function: Investigation of the Cognitive and Mood Effects of Mentha × Piperita L. Essential Oil With In Vitro Properties Relevant to Central Nervous System Function.” Nutrients 10, no. 8: 1029. 10.3390/nu10081029. 30087294 PMC6116079

[hup70005-bib-0032] Kennedy, D. O. , E. L. Wightman , J. L. Reay , et al. 2010. “Effects of Resveratrol on Cerebral Blood Flow Variables and Cognitive Performance in Humans: A Double‐Blind, Placebo‐Controlled, Crossover Investigation.” American Journal of Clinical Nutrition 91, no. 6: 1590–1597. 10.3945/ajcn.2009.28641. 20357044

[hup70005-bib-0033] Li, C. , W. Wang , Y. Wei , et al. 2025. “Association Between Cognitive Decline and Depression in Middle‐Aged and Older Adults: Findings From Six Large Cohorts in Different Countries.” Journal of Affective Disorders 371: 215–223. 10.1016/j.jad.2024.11.058. 39577500

[hup70005-bib-0034] López, V. , S. De Martin , M. P. Gómez‐Serranillos , M. E. Carretero , A. K. Jäger , and M. I. Calvo . 2010. “Neuroprotective and Neurochemical Properties of Mint Extracts.” Phytotherapy Research 24, no. 6: 869–874. 10.1002/ptr.3037. 19943334

[hup70005-bib-0035] McCombs, K. , B. Raudenbush , A. Bova , and M. Sappington . 2011. “Effects of Peppermint Scent Administration on Cognitive Video Game Performance.” North American Journal of Psychology 13, no. 3: 383.

[hup70005-bib-0036] Meamarbashi, A. 2014. “Instant Effects of Peppermint Essential Oil on the Physiological Parameters and Exercise Performance.” Avicenna Journal of Phytomedicine 4, no. 1: 72–78. https://pmc.ncbi.nlm.nih.gov/articles/PMC4103722/. 25050303 PMC4103722

[hup70005-bib-0037] Meamarbashi, A. , and A. Rajabi . 2013. “The Effects of Peppermint on Exercise Performance.” Journal of the International Society of Sports Nutrition 10, no. 1. 10.1186/1550-2783-10-15. PMC3607906 23517650

[hup70005-bib-0038] Moss, M. , S. Hewitt , L. Moss , and K. Wesnes . 2008. “Modulation of Cognitive Performance and Mood by Aromas of Peppermint and Ylang‐Ylang.” International Journal of Neuroscience 118, no. 1: 59–77. 10.1080/00207450601042094. 18041606

[hup70005-bib-0039] Moss, M. , R. C. Jones , L. Moss , R. Cutter , and K. Wesnes . 2016. “Acute Consumption of Peppermint and Chamomile Teas Produce Contrasting Effects on Cognition and Mood in Healthy Young Adults.” Plant Science Today 3, no. 3: 327. 10.14719/pst.2016.3.3.246.

[hup70005-bib-0040] Nawrot, J. , J. Gornowicz‐Porowska , J. Budzianowski , G. Nowak , G. Schroeder , and J. Kurczewska . 2022. “Medicinal Herbs in the Relief of Neurological, Cardiovascular, and Respiratory Symptoms After COVID‐19 Infection A Literature Review.” Cells 11, no. 12: 1897. 10.3390/cells11121897. 35741026 PMC9220793

[hup70005-bib-0041] Obrig, H. , and A. Villringer . 2003. “Beyond the Visible: Imaging the Human Brain With Light.” Journal of Cerebral Blood Flow and Metabolism 23, no. 1: 1–18. 10.1097/01.wcb.0000043472.45775.29. 12500086

[hup70005-bib-0042] Orhan, İ. E. , S. Aslan , M. Kartal , B. Šener , and K. H. C. Başer . 2008. “Inhibitory Effect of Turkish Rosmarinus Officinalis L. On Acetylcholinesterase and Butyrylcholinesterase Enzymes.” Food Chemistry 108, no. 2: 663–668. 10.1016/j.foodchem.2007.11.023. 26059146

[hup70005-bib-0043] Péronnet, F. , D. Mignault , P. Du Souich , et al. 2011. “Pharmacokinetic Analysis of Absorption, Distribution and Disappearance of Ingested Water Labeled With D2O in Humans.” European Journal of Applied Physiology 112, no. 6: 2213–2222. 10.1007/s00421-011-2194-7. 21997675 PMC3351614

[hup70005-bib-0044] Rapp, M. A. , M. Schnaider‐Beeri , M. Wysocki , et al. 2011. “Cognitive Decline in Patients With Dementia as a Function of Depression.” American Journal of Geriatric Psychiatry 19, no. 4: 357–363. 10.1097/JGP.0b013e3181e898d0. PMC3062696 20808140

[hup70005-bib-0045] Salm, S. , J. Rutz , M. van den Akker , R. A. Blaheta , and B. E. Bachmeier . 2023. “Current State of Research on the Clinical Benefits of Herbal Medicines for Non‐life‐threatening Ailments.” Frontiers in Pharmacology 14: 1234701. 10.3389/fphar.2023.1234701. 37841934 PMC10569491

[hup70005-bib-0046] Sato, H. , N. Yahata , T. Funane , et al. 2013. “A NIRS–fMRI Investigation of Prefrontal Cortex Activity During a Working Memory Task.” NeuroImage 83: 158–173. 10.1016/j.neuroimage.2013.06.043. 23792984

[hup70005-bib-0047] Scholey, A. , A. Ossoukhova , L. Owen , et al. 2010. “Effects of American Ginseng (Panax Quinquefolius) on Neurocognitive Function: An Acute, Randomised, Double‐Blind, Placebo‐Controlled, Crossover Study.” Psychopharmacology 212, no. 3: 345–356. 10.1007/s00213-010-1964-y. 20676609 PMC2952762

[hup70005-bib-0048] Silva, H. 2020. “Current Knowledge on the Vascular Effects of Menthol.” Frontiers in Physiology 11. 10.3389/fphys.2020.00298. PMC7154148 32317987

[hup70005-bib-0049] Sim, M. , S. Y. Kim , and Y. Suh . 2022. “Sample Size Requirements for Simple and Complex Mediation Models.” Educational and Psychological Measurement 82, no. 1: 76Â–106. 10.1177/00131644211003261. 34992307 PMC8725051

[hup70005-bib-0050] Talukdar, T. , J. H. Moore , and S. G. Diamond . 2013. “Continuous Correction of Differential Path Length Factor in Near‐Infrared Spectroscopy.” Journal of Biomedical Optics 18, no. 5: 056001. 10.1117/1.jbo.18.5.056001. 23640027 PMC4023650

[hup70005-bib-0051] Wang, L. , H. Ayaz , and M. Izzetoglu . 2019. “Investigation of the Source‐detector Separation in Near Infrared Spectroscopy for Healthy and Clinical Applications.” Journal of Biophotonics 12, no. 11. 10.1002/jbio.201900175. 31291506

[hup70005-bib-0052] Wei, E. T. 2020. “Improving Brain Power by Applying a Cool TRPM8 Receptor Agonist to the Eyelid Margin.” Medical Hypotheses 142: 109747. 10.1016/j.mehy.2020.109747. 32344288

